# A Diffusion Model Based on Network Intrusion Detection Method for Industrial Cyber-Physical Systems

**DOI:** 10.3390/s23031141

**Published:** 2023-01-19

**Authors:** Bin Tang, Yan Lu, Qi Li, Yueying Bai, Jie Yu, Xu Yu

**Affiliations:** 1 Qingdao Innovation and Development Base, Harbin Engineering University, Qingdao 266000, China; 2 Ship Science and Technology Co., Ltd., Harbin Engineering University, Qingdao 266000, China; 3 College of Information Science and Technology, Qingdao University of Science and Technology, Qingdao 266000, China; 4 Key Laboratory of Symbolic Computation and Knowledge Engineering of Ministry of Education, Jilin University, Changchun 130000, China

**Keywords:** diffusion model, intrusion detection, ICPS, imbalanced data, BiLSTM

## Abstract

Industrial Cyber-Physical Systems (ICPS) connect intelligent manufacturing equipment equipped with sensors, wireless and RFID communication technologies through data interaction, which makes the interior of the factory, even between factories, become a whole. However, intelligent factories will suffer information leakage and equipment damage when being attacked by ICPS intrusion. Therefore, the network security of ICPS cannot be ignored, and researchers have conducted in-depth research on network intrusion detection for ICPS. Though machine learning and deep learning methods are often used for network intrusion detection, the problem of data imbalance can cause the model to pay attention to the misclassification cost of the prevalent class, but ignore that of the rare class, which seriously affects the classification performance of network intrusion detection models. Considering the powerful generative power of the diffusion model, we propose an ICPS Intrusion Detection system based on the Diffusion model (IDD). Firstly, data corresponding to the rare class is generated by the diffusion model, which makes the training dataset of different classes balanced. Then, the improved BiLSTM classification network is trained on the balanced training set. Extensive experiments are conducted to show that the IDD method outperforms the existing baseline method on several available datasets.

## 1. Introduction

Cyber-Physical Systems (CPS) are the core of technology leading a new round of global industrial change, in which computing, communication, and control technologies are closely combined to realize the combination and coordination of computing and physical resources. Industrial Cyber-Physical Systems (ICPS) are the application of CPS in industry, which are widely used in industrial production, energy and power, traffic and driving, medical and health care, and urban construction. However, when the ICPS is increasingly connected to cyberspace, it has become one of the main targets of network attacks.

To avoid network attacks, researchers have made a lot of efforts to implement reliable intrusion detection systems for ICPS. Existing intrusion detection methods can be classified into three categories: (1) statistical-based methods [1,2,3], which detect network intrusion attacks by statistical and quantitative analysis. (2) machine learning-based methods [4,5,6,7], such as k-nearest neighbors (KNN), support vector machines (SVM), and decision trees (DT), etc. and (3) deep learning-based methods [8,9,10,11], such as deep belief networks (DBN), stacked autoencoders (SAE), convolutional neural networks (CNN), and recurrent neural networks (RNN).

However, real-world intrusion attacks contain multiple types, and there are some extremely sparse classes of intrusion attacks for which existing intrusion detection methods have poor classification performance. This is because the misclassification cost of rare class data in imbalanced data is much greater than the misclassification cost for prevalent class data. Imbalanced data makes it difficult for rare class data to be effectively learned by the classifier, which seriously affects the performance of the network intrusion detection model. Detecting effectively network intrusion attacks with rare classes makes administrators further adopt more accurate defense strategies, which can effectively ensure the network security of ICPS.

The diffusion model [12] has recently become a new state-of-the-art (SOTA) model in deep generative models, showing powerful performance in image generation tasks and multimodal generation tasks. Considering the powerful generative ability of the diffusion model, we apply the diffusion model to the task of generating rare class data for network intrusion detection and propose an ICPS Intrusion Detection Model Based on the Diffusion Model (DID). First, we gradually add noise to the rare class data during forward diffusion to obtain a fully Gaussian noise, and gradually denoise the Gaussian noise during reverse diffusion to construct the desired data from the noise. Then, the merged set of original and generated data is used as the balanced dataset on which the intrusion detection network based Bidirectional Long and short-term memory (BiLSTM) is trained. We conducted extensive experiments on two publicly available datasets to validate the effectiveness of our approach.

Our contributions are as follows:

- We use a diffusion model to equilibrate the rare class dataset. To the best of our knowledge, this is the first application of diffusion models to the study of ICPS network intrusion.
- We implemented network intrusion detection for ICPS using the BiLSTM model, which can accurately model and detects all types of network access traffic.
- We demonstrate the effectiveness of our approach for intrusion detection on imbalanced datasets through extensive experiments.

In Section 2, we sort out the ICPS intrusion detection work in recent years. In Section 3, we detail our proposed ICPS network intrusion detection approach based on the diffusion model. In Section 4, we conducted extensive experiments on two publicly available datasets. In Section 5, we present a summary and outlook of our work.

## 2. Related Work

### 2.1. Intrusion Detection

Intrusion detection models include three categories, namely, statistical-based methods machine learning-based methods, and deep learning-based methods.

For intrusion detection methods based on statistics. Manikopoulos et al. [13] proposed a statistical anomaly method, introduced a general anomaly and fault threshold system, and applied statistical preprocessing and simple neural network classification at the initial stage to detect network attacks and faults. The basis of the statistical model is to collect a large amount of training data, obtain the value range of each feature from the dataset, and divide the statistical interval, to determine the statistical measurement value of the system features, which is the foundation of early network intrusion detection [14]. However, statistical methods rely on a large number of known data, and cannot reflect the time sequence of the identified anomalies. At the same time, the setting of a threshold is one of the essential factors that affect the accuracy of the system.

As to machine learning-based methods. G Stein et al. [15] used genetic algorithms to select the input feature subset of the decision tree classifier to improve the detection rate and reduce the false positive rate in network intrusion detection. Roshan Chitrakar and Chuanhe Huang [16] proposed a semi-partition strategy by selecting and retaining non-support vectors of the current classification increment. This is an incremental SVM (CSV ISVM) algorithm based on candidate support vectors, which implements the entire process of incremental SVM classification. Robin Sommer and Vern Paxson [17] raised to apply the network intrusion detection system based on machine learning into the actual operating environment.

Owing to the dramatic increase in network traffic data volume and complexity, the traditional machine learning-based IDS with shallow structure is not suitable for the era of the Internet of Things with billions of devices. Accordingly, deep learning technology has been applied to the traditional NN architecture of deep neural networks (DNN). Mohammadi et al. [18] proposed an IDS model based on AE and Memetic algorithms. CNN is also a popular DNN with a hierarchical structure similar to digital images. The basic components of CNN are the convolution layer, pooling layer, and classification layer. Zhendong Wang et al. [19] proposed a knowledge distillation model based on a triple convolution neural network for network intrusion detection. Sheikhanden et al. [20] proposed a three-layer recurrent neural network (RNN) structure, which takes classification features as input and attack types as the output of RNN, as a misuse-based intrusion detection system. Because of the loop join, the past network activation state can be used for the current state to better represent time-related signals. Kim et al. [21] went deeply into the application of long and short-term memory (LSTM) in RNN-based IDS. However, most of the previously proposed methods ignore the balancing treatment of the rare class, resulting in poor performance in detecting rare classes of intrusion data [22].

### 2.2. Diffusion Model

Diffusion models surpass the original SOTA: Generative Adversarial Network (GAN) in image generation tasks and excel in many application areas such as computer vision, NLP, waveform signal processing, multimodal modeling, molecular graph modeling, time series modeling, adversarial purification, etc. Generative models are used in computer vision to handle various image recovery tasks including super-resolution, restoration, and panning [23,24,25], and in NLP to generate character-level text by discrete denoising diffusion probability models (D3PM) [26]. In addition, in terms of time series data generation, refs. [27,28] complemented missing values in time series data by diffusion models. In terms of data generation, the similar structure of time-series data and intrusion data, which are both one-dimensional data consisting of multiple features, inspired us to generate intrusion data with rare class data by diffusion models. To the best of our knowledge, diffusion model to network intrusion data for rare data balancing has not been reported in the literature.

## 3. Method

**Figure 1:**
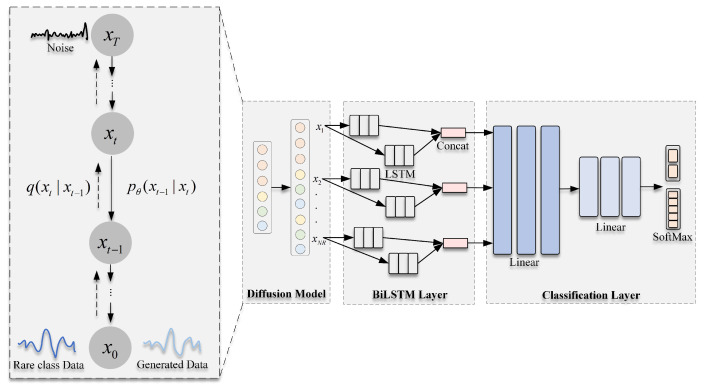
Intrusion detection flow chart based on the diffusion model.

In this paper, an intrusion detection model based on the diffusion model is proposed. As shown in Figure 1, the method consists of two modules: a data generation module based on the diffusion model and a classification module based on BiLSTM. The algorithm proposed in this paper is described in detail in the following.

### 3.1. Problem Description

In network intrusion detection scenarios, network access traffic can be divided into two categories: normal access (Normal), and intrusion attacks (Abnormal), where intrusion attacks also include Dos (Denial of Service Attacks), Probe (Probe Attacks), U2R (User to Root Attacks), R2L (Remote to Local Attacks), etc, each object is described by intrinsic, content, host-based and time-based features. Our goal is to predict the type of web network access traffic $X=\{x_{1},\cdots x_{n}\}$, which is a binary classification task Y={N,A}, where *n* is the number of data to be detected, *N* and *A* are abbreviations for the two categories of normal and abnormal respectively. To identify the type of network access traffic more precisely, it can be defined as a rare class classification task Y={N, Dos, Probe, U2R, R2L}.

In the data generation module, we generate rare class data based on the diffusion model to obtain a data-balanced expanded dataset. Specifically, given a set of rare class data $X^{R}=\left\{x_{1}^{R},\cdots x_{nr}^{R}\right\}$, the goal of the generation module is to learn a diffusion model that can generate a large number of data that can be used for downstream classification tasks $X^{R}=\left\{x_{1}^{R},\cdots ,x_{nr}^{R},\cdots ,x_{NR}^{R}\right\}$, where NR is much larger than nr.

In the intrusion detection module, the input data is the concatenation of the original and generated data $X=\left\{X^{N},X^{Dos},X^{Probe},X^{U2R},X^{R2L}\right\}\in R^{Num\times d}$, where Num=NN+ND+NP+NU+NR is the number of input data, NN, ND, NP, NU, and NR are the number of five types of access data, and *d* is the feature dimension. The task of the intrusion detection module is to learn a binary or multi-classification network that can accurately identify the types of network access traffic.

### 3.2. Feature Representation

The network access traffic contains various types of features ranging from textual to numerical. The text-based features mainly include protocol-type services and labels, etc., while the numerical features mainly include access length, counts and percentages, etc. Table 1 shows detailed information about these features, and next, we will give the representation and calculation of the features.

There are some features or labels in the dataset that are not represented by numerical values. To unify the encoding, we use the OrdinalEncoder() and LabelEncoder() functions of the preprocessing module in the sklearn library to unify the encoding of character-based features and labels, respectively. The data after the first numerical step is standardized, and Z-score standardization is one of the common methods for data processing. Assuming that the distribution of this data set approximately obeys Gaussian distribution, standardization is performed based on the mean and variance of the data, and the standardization formula is as follows.

(1) $$f^{\prime }=\frac{f-\bar{f}}{\sigma }$$

where *f* is the original data, $\bar{f}$ is the mean of the original data, σ is the standard deviation of the original data, and $f^{\prime }$ is the normalized data.

### 3.3. Data Generation Based on the Diffusion Model

The diffusion model usually consists of a forward process and a Reverse process. Given a data point $x_{0}∼q\left(x\right)$ sampled from a real data distribution, the forward process gradually corrupts $x_{0}$ to a standard Gaussian noise $x_{T}=N\left(0,I\right)$. For each forward step t∈[1,2,⋯,T], the noise perturbation is controlled by Equation (2), ${\left\{\beta_{t}\in \left(0,1\right)\right\}}_{t=1}^{T}$ to be a different variance scale. After completing the forward process once, the reverse denoising process gradually reconstructs the original data $x_{0}$ by learning the diffusion model $p_{\theta }$, through sampling $x_{T}$.

(2) $$q\left(x_{t}|x_{t-1}\right)=N\left(x_{t};\sqrt{1-\beta_{t}}x_{t-1},\beta_{t}I\right)$$

#### 3.3.1. Forward Diffusion Process

For a given data point $x_{0}∼q\left(x\right)$ sampled from a real data distribution, we define a forward diffusion process in which we add a small amount of Gaussian noise to the sample in *T* steps to produce a sequence of noisy samples $x_{1},\ldots ,x_{T}$. The step size is controlled by a variance ${\left\{\beta_{t}\in \left(0,1\right)\right\}}_{t=1}^{T}$. As the step size *t* becomes larger, the data sample $x_{0}$ gradually loses distinguishable features. Eventually, when T→∞,$x_{T}$ orresponds to an isotropic Gaussian distribution.

A nice property of the above procedure is that we can use the reparameterization trick to sample $x_{t}$ at any arbitrary time step *t* in closed form. Let $\alpha_{t}=1-\beta_{t}$ and $\bar{\alpha_{t}}=\prod_{i=1}^{t}\alpha_{i}$, i.e., $x_{t}=\sqrt{\bar{\alpha }}x_{0}+\sqrt{1-{\bar{\alpha }}_{t}\varepsilon }$. In this way, we can directly represent by $x_{0}$.

(3) $$q\left(x_{t}|x_{0}\right)=N\left(x_{t};\sqrt{{\bar{\alpha }}_{t}}x_{0},\left(1-{\bar{\alpha }}_{t}\right)I\right)$$

#### 3.3.2. Reverse Diffusion Process

By reversing the above process and sampling $q(x_{t-1}|x_{t})$, we can reproduce the real sample from the Gaussian noise input $x_{T}∼N\left(0,I\right)$. If $\beta_{t}$ is small enough, $q(x_{t-1}|x_{t})$ will also be Gaussian. It is worth noting that we cannot easily estimate $q(x_{t-1}|x_{t})$ because it requires using the entire data set, so we need to learn a model $p_{\theta }$ to approximate these conditional probabilities $p_{\theta }\left(x_{t-1}|x_{t}\right)$, to run the backpropagation process. According to the Markov rule representation, the process of reverse diffusion current time step *t* depends only on the previous time step t−1, so we have

(4) $$p_{\theta }\left(x_{t-1}|x_{t}\right)=N(x_{t-1};\mu_{\theta }\left(x_{t},t\right),\sum {}_{\theta }\left(x_{t},t\right)$$

Sample generation based on the diffusion model, that is, the diffusion process, manually adds a little noise until the data is pure Gaussian noise; reverse diffusion process to learn the distribution after the reversal, and gradually recover the sample data. This recovered sample data is the data we want to generate from fewer sample data.

#### 3.3.3. Parameter Training

The solution of the Markov process is usually sampled by the Monte Carlo method, and then we will evaluate the good or bad results of the sampling. Here we give the loss function directly:

(5) $$L_{t}=E_{x_{0},\varepsilon }[\frac{{\left(1-\alpha_{t}\right)}^{2}}{2\alpha_{t}\left(1-{\bar{\alpha }}_{t}\right)\left|\right|\sum_{\theta }{\left|\right|}_{2}^{2}}\left|\right|\varepsilon_{t}-\varepsilon_{\theta }\left(\sqrt{{\bar{\alpha }}_{t}}x_{0}+\sqrt{1-{\bar{\alpha }}_{t}}\varepsilon_{t},t\right){\left|\right|}^{2}]$$

Gaussian distribution is the noise predicted by the neural network model (for denoising), which can be viewed as $\varepsilon_{\theta }\left(x_{t},t\right)$. The core of Diffusion Models training is to take the learned Gaussian noise ε, between $\varepsilon_{\theta }$ the mean squared error MSE. Empirically, Ho et al. [12] found that it is better to train diffusion models with a simplified objective that ignores the weighting term, and proposed a simplified loss function:

(6) $$L_{t}^{simple}=E_{t∼\left[1,T\right],x_{0},\varepsilon_{t}}\left[|\right|\varepsilon_{t}-\varepsilon_{\theta }\left(\sqrt{{\bar{\alpha }}_{t}}x_{0}+\sqrt{1-{\bar{\alpha }}_{t}}\varepsilon_{t},t\right){\left|\right|}^{2}]$$

### 3.4. Classification Network

LSTM Network [29] is a variant of the RNN, which effectively stops the problem of gradient disappearance or gradient explosion by retaining important information and forgetting secondary information through memory state units. The BiLSTM network [30] model consists of a combination of a forward LSTM and a backward LSTM, aiming to encode feature units in the forward and backward directions, extract contextual semantic features and capture long-range dependencies.

In this paper, the BiLSTM model can well capture the long-range dependencies and contextual semantic information of the network access traffic. For example, it can establish long-range dependencies on both the basic characteristics of TCP connections and the content characteristics of TCP connections; it can also establish short-range dependencies on the number of accesses to system-sensitive files and the number of access control files in the content characteristics of TCP connections, and it can also mine the contextual semantic features in network access traffic.

Our improved BiLSTM model is trained in the depth of the features of network access traffic from both front and back directions and performs fine-grained network intrusion classification. The model is computed as shown below:

(7) $$\left\{\begin{array}{c} {\vec{h}}_{t}=f\left(w_{1}s_{t}+w_{2}{\vec{h}}_{t-1}\right)\\ {\overset{\leftarrow }{h}}_{t}=f(w_{3}s_{t}+w_{5}{\overset{\leftarrow }{h}}_{t+1})\\ y_{t}=g(w_{4}{\vec{h}}_{t}+w_{6}{\overset{\leftarrow }{h}}_{t}) \end{array}\right.$$

where ${\vec{h}}_{l}$ denotes the state of the forward LSTM layer at the moment, and ${\overset{\leftarrow }{h}}_{t}$ denotes the state of the backward LSTM layer at the moment *t*, corresponding to the contextual feature information of the network access traffic; $s_{t}$ denotes the unit vector of the input at the moment *t*; $w_{1}∼w_{6}$ denotes the weight parameter; *f* and *g* denote the activation function, usually using functions such as Relu; $y_{t}$ is the final output of the bidirectional long and short term memory network.

In this paper, the results of the generation module are used as the input of BiLSTM, which enables the training of a network intrusion model under balanced samples. The model combines contextual semantic knowledge to extract local features of network access traffic, safeguarding the dependence on long and short distances, and effectively improving the classification results of network access traffic on the web. Meanwhile, to prevent the overfitting phenomenon, this paper introduces the Dropout [31,32] mechanism to randomly select parameters for training.

The DNN classification network that implements the detection task has two hidden layers in it, and the DNN is computed with the following general formula:

(8) $$f\left(y_{t}\right)=\sigma \left(Wy_{t}+b\right)$$

## 4. Experiment and Results

In this paper, we use Pytorch deep learning framework to design neural network models, and the programming language is Python 3.7. Experimental configuration: Ubuntu 18.04 OS, 32GB RAM, NVIDIA RTX3060 8G GPU. This section evaluates the effectiveness of the proposed intrusion detection algorithm based on the diffusion model and detailed comparison experiments to further highlight the advantages of the detection model. Our main assessment questions are as follows.

RQ1: Is the detection performance of our method more outstanding compared to the current SOTA baseline methods?

RQ2: What is the classification performance of the method in this paper for rare class data?

RQ3: How effective is our treatment of the imbalance data problem based on the diffusion model?

### 4.1. Comparison Method

We propose an intrusion detection model based on the diffusion model (IDD). In particular, IDD includes a generation module and a classification detection module. Then, to evaluate the above-mentioned RQ1, RQ2, and RQ3, we select the existing state-of-the-art generation and classification models as the comparison method for the generation and detection modules, respectively. We use the hyperparameter settings for the baseline reported in their original paper or specified in their code unless otherwise noted.

#### 4.1.1. Generate Method

VAE [33]: Variational autoEncoder (VAE) is one of the generative models. The main goal of the methods is to generate new data from the learned distribution of objects.In 2014, Kingma et al. proposed this generative model that learns potential attributes and constructs new data from the probability distribution of the hidden variable space.

GAN [34]: Generative adversarial networks are an important generative model in the field of deep learning, where two networks (generator and discriminator) are trained at the same time and compete in a minimization-extreme algorithm. This adversarial approach avoids some of the difficulties of some traditional generative models in practical applications, cleverly approximates some unsolvable loss functions by adversarial learning, and has a wide range of applications in the generation of data such as images, videos, natural language, and music.

#### 4.1.2. Classification Models

CNN [35]: CNN emerged to handle the dense connectivity between DNN layers. CNN trains multiple layers by nonlinear mapping to classify high-dimensional input data into a set of classes in the output layer.

LSTM [29]: LSTM solves the gradient vanishing problem in vanilla RNN. It can learn long-term dependencies through the use of gating mechanisms. Each LSTM cell is equipped with a storage unit to save the old state.

AE [36]: A greedy hierarchical approach to feature learning combined with softmax regression as a classification layer is used to detect multi-class attacks.

DBN [37]: Deep Belief Network (DBN) consists of stacked RBMs, trained in a greedy hierarchical manner. In practice, DBNs are applied for dimensionality reduction, and when an additional discriminative layer is added, the DBN is also a stand-alone classifier.

### 4.2. Evaluation Indicators

To evaluate the performance of the method, we used metrics commonly used for intrusion detection: Check Accuracy Rate, Check Completeness Rate, F1-score, and Accuracy Rate, which are defined as follows:

$$Accuracy=\frac{\left|TN|+|TP\right|}{\left|FN|+|FP|+|TN|+|TP\right|}$$

$$\mathrm{Precision}=\frac{\left|TP\right|}{\left|FP|+|TP\right|}$$

$$\mathrm{Recall}=\frac{\left|TP\right|}{\left|FN|+|TP\right|}$$

$$F1-measure=\frac{2\times \mathrm{Recall}\times \mathrm{Precision}}{\mathrm{Recall}+\mathrm{Precision}}$$

where TP (true positive) is the number of attacks correctly classified, FP (false positive) is the number of normal visits incorrectly classified as attacks, TN (true negative) is the number of normal visits correctly classified, and FN (false negative) is the number of normal visits incorrectly classified. Accuracy is the ratio of correctly predicted attacks to the total predicted attacks. Recall (also known as detection rate) is the ratio of correctly predicted attacks to total attacks. The F1-score can be interpreted as a weighted summed average of the accuracy and the completeness of the check.

### 4.3. Dataset

#### 4.3.1. Data Set Introduction

NSL-KDD dataset [38]: To make deep learning algorithms work better on KDD Cup99, researchers created the NSL-KDD dataset based on the KDD Cup99 dataset in 2000. This dataset removes duplicate records from KDD Cup99 and reduces the amount of data. NSL-KDD contains the basic records and data characteristics of the KDD Cup99 dataset, and the identified attack categories are all the same as in the KDD Cup99 dataset.

UNSW-NB15 dataset [39]: The dataset was designed and completed by the Australian Cyber Security Centre Laboratory in 2015 and is an open-source dataset. The dataset is trained using the IXIA traffic generator to simulate as realistic an attack environment as possible, based on publicly available vulnerability information technology on the CVE website. The dataset has 49 feature attributes, similar to the KDD Cup99 dataset, including 5 traffic features, 13 basic features, 8 content features, 9 temporal features, 12 other features, and 2 marker features.

#### 4.3.2. Data Pre-Processing

For the dataset used in the experiment, we take NSL-KDD as an example, which is a new dataset improved by Mahbod Tavallaee et al. for the KDD_cup99 dataset, and the NSL-KDD dataset includes four sub-datasets, KDDTrain+, KDDTest+, KDDTrain+_20Percent, and KDDTest_21. where KDDTrain+_20Percent contains only the top 20% of KDDTrain+ data. We use KDDTrain+_20Percent as the training set and KDDTest+ as the test set. The five classification labels in the dataset are Normal, Probing, R2L, U2R, and Dos. For datasets without pre-segmentation, to ensure consistent data distribution between the training and test sets, we sampled 70% of the data from each classification for training and 30% of the data for the test set.

**Figure 2:**
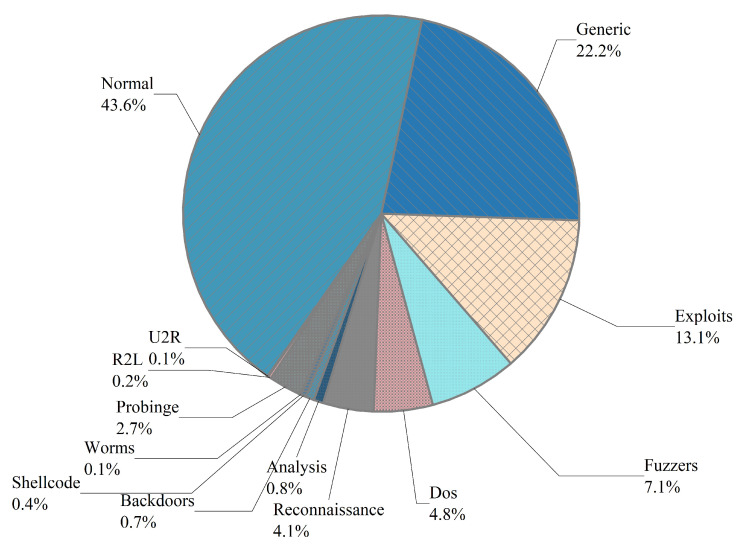
Distribution of all categories of data on the NSL-KDD and UNSW-NB15 datasets.

To verify that the intrusion detection method based on the diffusion model proposed in this paper can effectively expand the imbalanced dataset and achieve the detection of the type of network access traffic, all the rare class network access traffic generated by the diffusion model in this paper until the number is the same as the average number of attack data (For example, on the NSL-KDD dataset, we expand Dos, Probe, R2L, and U2R all to 2936 entries until they are the same as the average number of attack data). We aim to have the same proportion of all types of data in the dataset and thus train the classification detection network on a balanced dataset. The type distribution of the dataset is shown in Table 2 and Figure 2.

#### 4.3.3. Parameter Setting

**Figure 3:**
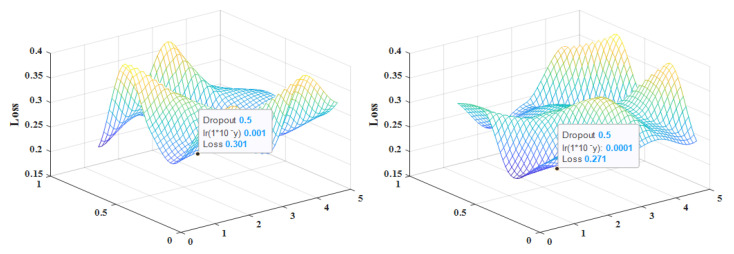
Parameter tuning situation. (NSL-KDD & UNSW-NB15).

Different hyperparameter settings affect the model convergence speed and experimental results. We determine the optimal parameters by ten-fold cross-validation and use the best parameters to retrain the training set. The configuration of hyperparameters for this experiment is shown in Table 3, and the parameter tuning is shown in Figure 3.

Where the # in the table is the feature dimension of the dataset, which can be set according to the actual needs, and the * in the table is set according to the actual demand, if n classification tasks are performed then set to n.

It is worth noting that each sub-model of the algorithm has different optimal combinations of parameters for different training sets, therefore, the parameters should be re-tuned for new training data to obtain the best performance of the algorithm.

### 4.4. Results Comparison

**RQ1: Is the detection performance of our method more outstanding compared to the current state-of-the-art baseline methods?**

In this paper, the diffusion model is applied to the generation of web access traffic data, and the detection model is further trained based on a balanced dataset. To verify the effectiveness of the method in this paper, we conduct comparison experiments with existing state-of-the-art intrusion detection methods and restore the detection model of the reference in detail, respectively. These methods include CNN, LSTM, BiLSTM, and DBN. We use the hyperparameter settings specified in their original papers for these comparison methods, and the optimal parameters from multiple experiments are used for the parameters not mentioned. The experimental results are shown in Table 4.

By comparing the individual metrics of the five methods in Table 4 on the two data sets, it can be seen that the model in this paper outperforms the baseline method in all metrics. BiLSTM can capture long-range dependencies and contextual semantic information of network access traffic in both directions, and also mine the contextual semantic features in network access traffic. Our method is based on the BiLSTM model, to which a rare class data generation module is added to construct a balanced dataset. The BiLSTM model trained on the constructed dataset performs significantly better than the BiLSTM model trained on the original data, demonstrating the effectiveness of our approach for network intrusion detection with the imbalanced dataset. The other methods still achieved performance metrics of more than 85%, although they were not processed for rare class data generation. This is because the percentage of rare class data is very low on each dataset (e.g., only 0.89% of U2R data in the NSL-KDD test set), and their misclassification effect has an insignificant impact on the overall performance of the model. In terms of accuracy metrics, our method and BiLSTM have a significant improvement compared to the other three methods, proving that BiLSTM has a strong enough feature extraction ability and classification ability.

**RQ2: What is the classification performance of the method in this paper for rare class data?**

In this paper, a diffusion model is used to generate the rare class data, and all two data sets are balanced so that the same amount of web access traffic data is available for each category. To verify the performance of the method in this paper for rare class classification, we conducted comparative experiments on two datasets. where *p*-value is the probability that the observed value or a value more extreme than the observed value will occur if the null hypothesis holds. In this experiment, the p-value indicates whether the detection performance of the balanced dataset under the condition that the classification network does not change is better than other methods, i.e., *p*-value < 0.5.

The results in Table 5 show that for the rare class classification task, the model in this paper has a significant advantage in the rare class (R2L and U2R) with an accuracy rate of 65%. The model performs significantly better than the other comparison methods in terms of significance testing. This also demonstrates the effectiveness of the diffusion model-based rare class generation module.

**RQ3: How effective is our treatment of the imbalanced data problem based on the diffusion model?**

**Figure 4:**
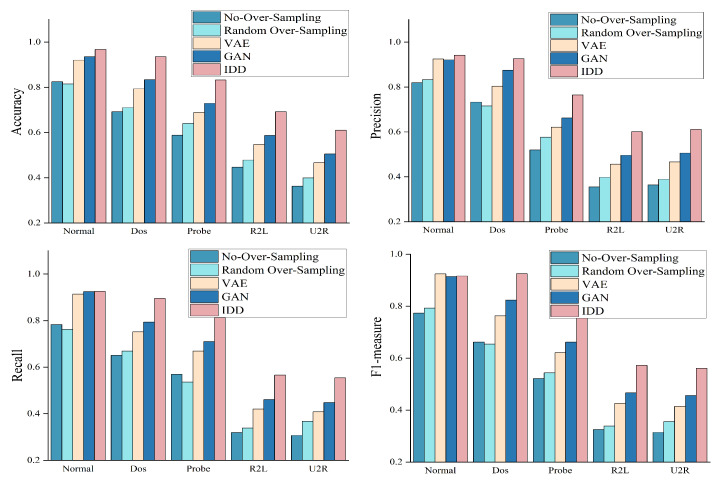
Classification performance of BiLSTM on data generated by different generative models (NLS-KDD dataset).

**Figure 5:**
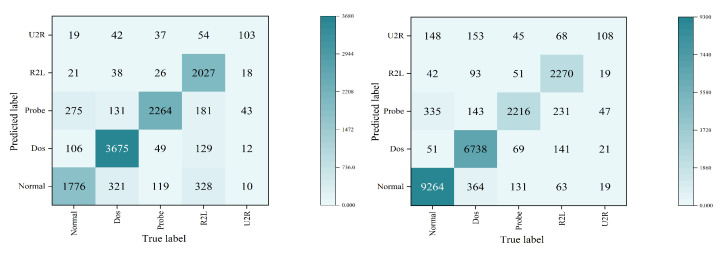
IDD confusion matrix (KDDTest+ and KDDTest-21).

In this section, Random Over-Sampling, VAE, GAN, and Ours are used to generate the rare class data, which are compared to the NLS-KDD dataset to verify the effectiveness of the diffusion model to generate rare class data. It is worth noting that the classification network uniformly uses the BiLSTM model. The experimental results are shown in Figure 4 and Figure 5. In Figure 4, we denote BiLSTM without Over-Sampling as No-Over-Sampling, BiLSTM with Over-Sampling as Over-Sampling, BiLSTM with VAE as VAE, BiLSTM with GAN as GAN, BiLSTM with diffusion model as IDD.

From the experimental results in Figure 4, it can be seen that the original dataset without any processing has a very poor performance in the classification of rare class data, and the accuracy of detection is only 30%. This is because the imbalanced data makes it difficult for rare class samples to be effectively learned by the classifier, making the model less accurate in detecting the rare class data. And because the number of data of intrusion attack Dos is relatively large, reaching almost the same number as Normal data, the model achieves a high level of detection accuracy for them. From the experimental results in Figure 5, IDD can identify most of the rare class of intrusion detection attacks, and the number of misclassifications is kept very low.

The BiLSTM-based classification network performs significantly better on the dataset generated by the diffusion model than other methods. The main reasons are as follows. Compared to generative models, the oversampling approach adds only duplicate data to the original dataset, leading to overfitting problems in model training and making the model perform poorly on test data. The training process of GAN model is unstable and the generated samples are not sufficiently diverse [40]. VAE relies on surrogate loss and the performance is also unsatisfactory [41]. The diffusion model is essentially a combination of GAN and VAE [42], which on the one hand draws on the idea of a single training target of the GAN model, and on the other hand exploits variational inference of the VAE model, thereby generating samples with better diversity.

## 5. Conclusions

For the network intrusion detection of ICPS, we propose an ICPS Intrusion Detection system based on the Diffusion model (IDD). The IDD model contains a rare class sample generation module and a classification module. Compare with traditional Oversampling and sample generation methods, the diffusion model used in this paper can model data distribution more accurately and generate high-quality balanced dataset. In the classification module, the BiLSTM-based module can extract richer high-level features on a balanced dataset to improve the performance of intrusion detection. Two comparative experiments demonstrate that IDD’s intrusion detection performance outperforms that of existing state-of-the-art methods on two public datasets. In detail, IDD not only improves the overall anomaly detection performance but also improves the detection performance on each attack class (i.e., Normal, DoS, Probe, R2L, and U2R), especially on R2L and U2R attacks. This indicates that IDD has better performance improvement for extremely rare class. However, the diffusion model has an inherent drawback of a large number of sampling steps and a long sampling time. In subsequent work, we hope to speed up the diffusion process while improving the sampling quality and the speed of generating rare class data.

## Figures and Tables

**Table 1:** Description of some features.

Feature Name	Description	Value Type	Ranges
Protocol Type	Protocol used in the connection	Textual	
Num Failed Logins	Count of failed login attempts	Numerical	0–4
Root Shell	1 if root shell is obtained; 0 otherwise	Numerical	0, 1
Same Srv Rate	The percentage of connections that have activated the flag REJ	Numerical	0, 1
Class	Classification of the traffic input	Textual	
Difficulty Level	Difficulty level	Numerical	0–21

**Table 2:** The details of the NSL-KDD dataset.

Type	Train_Original		Test		Train_Generated	
	Records	Percentage	Records	Percentage	Records	Percentage
Normal	13,449	53.38%	9711	43.07%	13,449	39.07%
Dos	9234	16.65%	7458	33.08%	9234	26.82%
Prob	2289	9.08%	2421	10.73%	2936	8.53%
R2L	209	0.83%	2754	12.21%	2936	8.53%
U2R	11	0.04%	200	0.89%	2936	8.53%
Total	25,192		22,544		34,427	

**Table 3:** Hyperparameter Configuration.

BiLSTM Model			Classification Model		
Layer	Type	Output Shape	Layer	Type	Output Shape
1	Embedding	#	8	Linear	128, 64
2	BiLSTM	#, 64	9	Relu	64, 64
3	Relu	64, 64	10	Dropout	64, 64
4	Dropout	64, 64	11	Linear	64, 32
5	BiLSTM	64, 128	12	Relu	32, 32
6	Relu	128, 128	13	Dropout	32, 32
7	Dropout	128, 128	14	Full connect	32, *

**Table 4:** The detection performance of IDD and baseline methods on different data sets.

Dataset	Model	Accuracy	Precision	Recall	F1-measures
NSL-KDD	CNN	0.739	0.866	0.784	0.790
	DBN	0.763	0.879	0.806	0.811
	LSTM	0.812	0.903	0.813	0.836
	BiLSTM	0.866	0.922	0.832	0.877
	Ours	0.896	0.931	0.853	0.894
UNSW-NB15	CNN	0.746	0.857	0.776	0.781
	DBN	0.771	0.871	0.797	0.802
	LSTM	0.820	0.893	0.804	0.826
	BiLSTM	0.874	0.912	0.823	0.867
	Ours	0.904	0.921	0.843	0.884

**Table 5:** Performance of IDD on rare class classification (v: value; *p*-v: *p*-value).

Datasets	Type	Accuracy		Precision		Recall		F1-measure	
		v	*p*-v	v	*p*-v	v	*p*-v	V	*p*-v
NSL-KDD	Normal	0.937	*p* < 0.5	0.942	*p* < 0.5	0.926	*p* < 0.5	0.916	*p* < 0.5
	Dos	0.935		0.926		0.895		0.925	
	Probe	0.832		0.765		0.813		0.765	
	R2L	0.692		0.601		0.566		0.572	
	U2R	0.610		0.611		0.554		0.561	
UNSW-NB15	Normal	0.943	*p* < 0.5	0.938	*p* < 0.5	0.933	*p* < 0.5	0.932	*p* < 0.5
	Generic	0.974		0.963		0.912		0.912	
	Exploits	0.873		0.885		0.869		0.845	
	Fuzzers	0.824		0.794		0.839		0.827	
	Dos	0.778		0.698		0.761		0.753	
	Backdoors	0.696		0.703		0.747		0.689	

## Data Availability

Not applicable.
